# Plasma Proteomic Profiling Reveals Alterations in Coagulation, Complement, and Extracellular Matrix Pathways in Orthostatic Hypotension

**DOI:** 10.1016/j.jacadv.2026.103116

**Published:** 2026-08-11

**Authors:** Madeleine Johansson, Ákos Végvári, Gunnar Engström, Artur Fedorowski

**Affiliations:** a Department of Clinical Sciences, Lund University, Malmö, Sweden; b Department of Medical Biochemistry and Biophysics, Karolinska Institutet, Stockholm, Sweden; c Department of Cardiology, Karolinska University Hospital, Stockholm, Sweden; d Department of Medicine, Karolinska Institute, Stockholm, Sweden

**Keywords:** biomarkers, blood clotting, coagulation, orthostatic hypotension, proteomics

**What is the clinical question being addressed?**

Which biological and molecular processes contribute to the pathophysiology of orthostatic hypotension?

**What is the main finding?**

Orthostatic hypotension is associated with alterations in coagulation, complement, and extracellular matrix pathways, pointing to molecular effects beyond blood pressure regulation and broader vascular and inflammatory phenotypes.

Orthostatic hypotension (OH) affects one-fifth of older adults and is an important clinical marker of autonomic nervous system dysfunction.^1^ It is associated with increased risks of syncope, adverse cardiovascular outcomes, and mortality, independent of traditional risk factors, yet it remains frequently underdiagnosed in routine practice. Almost 40% of patients with moderate-to-severe OH lack an identifiable etiology, highlighting persistent gaps in understanding the underlying pathophysiology of OH.^2^

Plasma proteomics is useful to characterize different biomarker profiles. Previous studies from our research group using Proximity Extension Assay, have linked OH to plasma biomarkers involved in immune regulation, vascular inflammation, and atherosclerosis,^3^^,^^4^ but broader, untargeted proteomic approaches remain limited. To address this gap, we investigated the plasma proteomic signature of OH using isobaric chemical labeling and liquid chromatography–tandem mass spectrometry, which offers better sensitivity and reproducibility than antibody-based assays.^5^

We performed a case-control study, including 100 patients (aged 64 ± 17 years, age range 20-92 years, 42% men) randomly selected from the Syncope Study of Unselected Population in Malmö study with OH verified by a head-up tilt test according to current guidelines, defined as a systolic blood pressure (BP) drop of ≥20 mmHg and/or diastolic BP drop of ≥10 mmHg within 3 min of standing,^1^ compared with 100 randomly selected controls (aged 62 ± 4 years, age range 52-65 years, 42% men) from the general population enrolled in the Swedish CArdioPulmonary bioImage Study Malmö with a negative orthostatic standing test. Only individuals with complete orthostatic BP and demographic data, and available plasma samples were included. The Syncope Study of Unselected Population in Malmö and Swedish CArdioPulmonary bioImage Study studies were approved by The Regional Ethical Review Boards (No. 82/2008; 2010-228-31M; 2016-511-31M; 2017-14-31; 2016/1031). All study participants provided written informed consent.

Plasma proteins were identified by deep proteomic profiling using isobaric chemical labeling method and liquid chromatography-tandem mass spectrometric analysis. Plasma was obtained by centrifugation of blood immediately after sampling into EDTA tubes. The samples remained frozen at −80 °C until analysis without previous thawing. Differential expression testing and Benjamini-Hochberg method were applied to quantified proteins with 100% completeness. A sensitivity analysis was performed using linear regression analysis adjusted for age and cohort, as well as corrected for multiple testing using the Benjamini-Hochberg method.

A total of 569 plasma proteins were quantified in all study participants, of which 15 were significantly dysregulated in OH patients compared with controls using a cutoff fold change of 1.2 and false discovery rate (FDR; q-value) of <0.05. Nine plasma proteins were significantly upregulated, and 6 plasma proteins were significantly downregulated in OH (all *P* < 0.05) (Table 1).

**Table 1:** Significantly Dysregulated Proteins in Orthostatic Hypotension

Protein Name	UniProt ID	Gene Name	Fold Change (95% CI)	*P* Value
Upregulated Proteins				
Apolipoprotein A-II	P02652	APOA2	1.17 (1.09-1.35)	0.006
Ceruloplasmin	P00450	CP	1.28 (1.15-1.40)	<0.001
Complement component C8 beta chain	P07358	C8B	1.24 (1.41-2.04)	0.001
Fibrinogen alpha chain	P02671	FGA	1.26 (1.13-1.39)	<0.001
Fibrinogen beta chain	P02675	FGB	1.29 (1.10-1.35)	0.001
Fibrinogen gamma chain	P02679	FGG	1.29 (1.12-1.38)	0.001
Fibronectin	P02751	FN1	1.59 (1.42-1.93)	<0.001
Ficolin-3	O75636	FCN3	1.45 (1.29-1.72)	<0.001
Vitamin D-binding protein	P02774	GC	1.91 (1.63-2.01)	<0.001
Downregulated Proteins				
Coagulation factor X	P00742	F10	0.72 (0.70-0.88)	0.002
Inter-alpha-trypsin inhibitor heavy chain H1	P19827	ITIH1	0.71 (0.69-0.86)	0.002
Inter-alpha-trypsin inhibitor heavy chain H2	P19823	ITIH2	0.72 (0.70-0.87)	0.002
Lipopolysaccharide-binding protein	P18428	LBP	0.45 (0.44-0.57)	<0.001
Mannan-binding lectin serine protease 1	P48740	MASP1	0.69 (0.65-0.85)	<0.001
Prothrombin	P00734	F2	0.71 (0.68-0.86)	<0.001

UniProt ID refers to accession numbers obtained from the UniProt Database, which is the world’s leading high-quality, comprehensive resource of protein sequence and functional information. Deep proteomics data of significantly dysregulated proteins in OH using a cutoff log2 fold change set to 1.2 and false discovery rate (q-value) of <0.05.

APOA2 = Apolipoprotein A-II; C8B = Complement component C8 beta chain; CP = Ceruloplasmin; F2 = Prothrombin; F10 = Coagulation factor X; FCN3 = Ficolin-3; FGA = Fibrinogen alpha chain; FGB = Fibrinogen beta chain; FGG = Fibrinogen gamma chain; FN1 = Fibronectin; GC = Vitamin D-binding protein; ITIH1 = inter-alpha-trypsin inhibitor heavy chain H1; ITIH2 = inter-alpha-trypsin inhibitor heavy chain H2; LBP = lipopolysaccharide-binding protein; MASP1 = mannan-binding lectin serine protease 1.

To identify significant protein-protein interactions (PPIs), we performed a pathway analysis of 15 significantly dysregulated plasma proteins in OH using STRING. The pathway provided a network of upregulated (PPI enrichment *P* value: <1.0e-16) and downregulated proteins (PPI enrichment *P* value: 1.09e-09). The strongest network of upregulated proteins was observed between all three fibrinogen chains (alpha, beta, and gamma). The strongest network of downregulated proteins was found between prothrombin and coagulation factor X.

In the gene ontology protein enrichment analysis, we observed the distribution of the top 10 gene ontology biological processes identified by proteomics in OH. The strongest PPI network in OH was associated with blood clotting and fibrin formation, followed by fibrinolysis and zymogen activation.

Overall, our findings suggest that OH is associated with systemic biological changes extending beyond classical autonomic dysfunction, involving vascular, inflammatory, and hemostatic pathways, and a distinct plasma proteomic footprint, characterized by changes in coagulation factors, complement pathway proteins, and extracellular matrix components.

We observed several key proteins associated with the coagulation cascade that were significantly dysregulated in patients with OH. Notably, prothrombin and coagulation factor X were downregulated, whereas fibronectin and fibrinogen chains (alpha, beta, and gamma) were upregulated. We hypothesized that this may indicate a paradoxical state in which substrates for fibrin formation are elevated in OH, yet key enzymes required for thrombin generation are reduced. We hypothesize that the latter may suggest a biological response to increased fibrin formation. Interestingly, we have previously reported elevated levels of thrombomodulin in OH,^3^ suggesting an endothelial-coagulation crosstalk.

In OH, resistance vessels in the vasculature of the abdomen and lower extremities fail to constrict during orthostasis, leading to inadequate peripheral vascular resistance and excessive venous pooling. We speculate that reduced thrombin generation may impair the vasoconstrictive responses during orthostasis, contributing to the inadequate vasoconstrictive compensation observed in OH. On the contrary, elevated fibrinogen and fibronectin levels may reflect chronic endothelial stress or low-grade inflammation. These findings are in line with our previous research, where we observed upregulation of proteins related to immune activation and vascular inflammation, such as matrix metalloproteinase 7, T-cell immunoglobulin and mucin domain 1,^3^ and immunoglobulin-like transcript 3.^4^ All of which are associated with exacerbated endothelial dysfunction and coagulation disturbances.

We further observed alterations in the complement pathway, with upregulation of ficolin-3 and complement C8 beta chain, and downregulation of mannan-binding lectin serine protease 1 and lipopolysaccharide-binding protein, suggesting an imbalance within particularly the lectin complement pathway, and activation of the innate immune system and chronic low-grade inflammation in OH. mannan-binding lectin serine protease 1 plays an important role in clotting and fibrin formation, and reduced levels are associated with impaired immune response and coagulation disturbances. This proinflammatory and prothrombotic vascular environment may in turn be promoted by dysregulation of the complement system and its interactions with the coagulation system.

Similarly, inter-alpha-trypsin inhibitor heavy chain H1 and H2 were downregulated in OH, suggesting impaired regulation of protease activity, and eventually increased inflammation and tissue remodeling. Reduced inter-alpha-trypsin inhibitor heavy chain H1 and H2 levels have been linked to endothelial fragility and increased vascular compliance. In OH, excessive vascular compliance may exacerbate venous pooling and impair the ability to maintain BP on standing. We speculate that the concurrent elevation of fibronectin may represent a compensatory response to extracellular matrix degradation, consistent with studies showing fibronectin upregulation in conditions of endothelial injury and vascular remodeling.

We further observed upregulation of proteins associated with vascular injury, hypoperfusion, and chronic inflammatory states, including increased levels of vitamin D–binding protein and ceruloplasmin, which suggests a broader systemic response that may involve chronic low-grade vascular stress and extracellular matrix remodeling in OH. This is in line with our previous study, where we noted elevated levels of markers of endothelial injury and vascular dysfunction in OH, such as thrombomodulin.^3^

Taken together, our findings suggest that OH is a condition that extends beyond autonomic failure and involves protein signatures associated with altered coagulation and fibrinolysis, dysregulated complement, and innate immune pathways, as well as extracellular matrix remodeling and endothelial stress. Although autonomic failure remains a key driver of OH, our results suggest that systemic vascular and inflammatory processes may amplify susceptibility to orthostatic BP drops and increased cardiovascular risk associated with OH. The proteomic alterations identified in this study have several potential clinical implications that may help explain why patients with OH exhibit limited response to conventional therapies that target only autonomic mechanisms. Although the current cross-cohort design warrants independent replication with matched sample handling to confirm the disease-specificity of the identified associations, integrating molecular profiling with clinical assessment may ultimately support more personalized approaches aimed at identifying patients at heightened cardiovascular risk, as well as improving orthostatic BP regulation and prognosis.

## Funding Support and Author Disclosures

This study was funded by the Bundy Academy. The main funding body of The Swedish CArdioPulmonary bioImage Study (SCAPIS) is the Swedish Heart and Lung Foundation. The authors have reported that they have no relationships relevant to the contents of this paper to disclose.
